# Reinforcement Effect of Different Fibers on Asphalt Mastic

**DOI:** 10.3390/ma15238304

**Published:** 2022-11-23

**Authors:** Tingting Xie, Kang Zhao, Linbing Wang

**Affiliations:** 1 National Center for Materials Service Safety, University of Science and Technology Beijing, Beijing 100083, China; 2 School of Environmental, Civil, Mechanical and Agricultural Engineering, University of Georgia, Athens, GA 30602, USA

**Keywords:** asphalt mastic, lignin fiber, carbon fiber, rheology, burgers model

## Abstract

Fiber materials as an asphalt mixture additive and stabilizer can effectively improve the performance index of asphalt pavement. In this study, lignin and carbon fiber were used as modifiers to study their effects on the road performance of asphalt mastic. Based on the frequency sweep, linear amplitude sweep (LAS) and multi-stress creep recovery (MSCR) experiments were conducted to test the high-temperature rutting and medium-temperature fatigue resistance of asphalt mastic with different fiber incorporation and low-temperature performance tests based on bending beam rheometer (BBR). The results indicate that adding fibers increased the stiffness of the asphalt mastic, and the modification effect of lignin fibers was better than that of carbon fibers. Meanwhile, the characteristic flow index of the asphalt mastic gradually increased with the increase in temperature, indicating that it gradually became a near-Newtonian fluid at higher temperatures. The addition of fibers also improved the high temperature rutting resistance of the asphalt mastic but did not have an advantageous effect on fatigue and low temperature cracking resistance. Additionally, the fitting results of the four-parameter Burgers model show that the use of fiber modification decreases the proportion of elasticity and viscous creep compliance but increases the delayed elasticity part.

## 1. Introduction

Different fibers have been used in asphalt binders and mix to address mechanical performance issues. Fibers provide this material with higher modulus, resistance, durability, deformability, and excellent ductility [1]. In addition, fibers have been used as reinforcement for polymer matrices in other industries [2,3]. These fibers give stiffness and strength to the composite, thus allowing the matrix to better transfer loads between fibers. Similarly, adding fibers to asphalt can improve the performance of the mix and contribute to sustainability by extending its service life and reducing road maintenance. Many studies have been conducted on fiber-reinforcement asphalt mixtures or mastics [4,5,6,7]. There are two main categories of fibers: synthetic fibers and natural fibers [8]. Synthetic fibers include polyester, carbon, and glass fibers. It has been demonstrated that applying synthetic fibers to asphalt mixtures can improve their fatigue life [9,10,11]. Wu et al. studied the voids and low-temperature performance of asphalt mixes with different polyester fiber contents. It was discovered that with the increase of polyester fiber content, both voids and connected voids decreased first and then increased, with the best performance in low-temperature crack resistance produced given a 0.4% polyester fiber content [12]. Qin et al. characterized the performance of different fibers (including basalt fibers, polyester fibers, and lignin fibers) on asphalt biners, and the results showed that asphalt binders reinforced with basalt fibers showed the best overall performance [13]. In light of moisture damage and low temperatures, Khater et al. evaluated the efficiency of lignin fiber and glass fiber-modified asphalt mixes. Fibers significantly improved the water resistance, low-temperature stability, and quality of asphalt mixtures [14]. Fu et al. evaluated the fracture resistance of different fiber-modified asphalt mixes based on acoustic emission techniques and found that the incorporation of fibers greatly improved the strength of the control asphalt mixture and that steel fiber-reinforced asphalt mixes exhibited more favorable fracture resistance than glass fiber-reinforced asphalt mixes and basalt fiber-reinforced asphalt mixtures [15]. Abtahi et al. investigated the strengthening effect of asphalt mixtures incorporated with both glass and polypropylene fibers in the range of 0.05% to 0.2% by weight of aggregate for glass fibers and 2% to 6% by weight of asphalt for polypropylene fibers, and proposed an optimum amount of 0.1% glass fibers in combination with 6% polypropylene fibers [16].

Since natural fibers usually exhibit considerable mechanical properties and are more environmentally friendly and cheaper than synthetic fibers, they are often used to reinforce asphalt mixtures such as cotton, hemp, wool, and silk fibers. Chen et al. conducted a systematic study and analysis of several types of fiber-modified asphalt mixes through penetration tests and dynamic shear tests and pointed out that the fiber surface area should be taken into account when increasing asphalt amount and when mixing asphalt mixes in order to meet the needs of asphalt-coated fibers [17]. Miao et al. investigated the effect of interfacial properties on the performance of fiber-reinforced asphalt. They found that fiber reinforcement was significantly more effective when fiber surface energy was high, and the fiber surface energy correlated positively with the fiber reinforcement effects [18]. Stone Matrix Asphalt (SMA) was investigated by Zhang et al. to determine its rheological behavior and strengthening mechanism. They found that the fibers in the asphalt mastic were well strengthened and that the creep recovery rate of asphalt mastic increased significantly, while the residual creep value decreased under high stress [19]. Research from Noorvand et al. found that fiber-reinforced asphalt mixes with higher micro fibrillation had a better dispersion uniformity and were more resistant to high-temperature rutting [20]. Tanzadeh et al. modified asphalt binders with 4.5% styrene-butadiene-styrene (SBS) and 2% and 4% nano-silica to improve the performance properties of the modified porous asphalt mixes by adding 0.5% and 1% lime powder and blended synthetic fibers to 0.4% and 0.5% of the asphalt mix as filler types, resulting in weight loss, while improving tensile strength and rutting resistance [21]. Liu and Xia et al. found that bamboo fibers outperformed lignin fibers regarding high-temperature stability, low-temperature crack resistance, and moisture stability of asphalt mixtures with good road properties [22,23]. Sheng et al. reported that adding bamboo fibers to asphalt mixes improved their water damage resistance, rutting resistance, and cracking resistance at low temperatures. It was determined from Marshall mix design calculations that bamboo fiber content should be 0.2–0.3% for dense grade asphalt (DG) and 0.4% for stone matrix asphalt (SMA) [24]. Yu et al. found that bamboo fiber incorporation improved the stability and tensile strength of the asphalt mixture, and SEM images revealed a strong bond between the fiber modified asphalt mixture and the asphalt binder [25].

As fiber reinforcement technology is currently used mainly in asphalt mixtures, and asphalt mastic is an essential binder, the interaction between it and fibers has been less studied. Most studies were conducted on a single fiber type, and not enough research has been conducted on the reinforcing effect of different fiber types. Therefore, two different fibers, carbon fiber and lignin fiber, were selected for this study to select a better-performing fiber and promote its application in asphalt pavements. Frequency scan (FS) tests were used to evaluate the linear viscoelastic rheological properties; fatigue properties were evaluated using linear amplitude scan (LAS) tests coupled with viscoelastic continuum damage (VECD) theory; rutting resistance was evaluated using multiple stress creep recovery (MSCR) tests; bending beam rheometer (BBR) tests were used to evaluate the crack resistance of asphalt mastics; and the Burgers model was used to analyze the viscoelastic composition of the fiber-modified asphalt mastic.

## 2. Materials and Methods

### 2.1. Materials

#### 2.1.1. Asphalt Binder

The asphalt binder (penetration grade of pen-70) provided by Beijing Changping Aphalt Plant was selected as the base asphalt for this study. The main physical characteristics of the base asphalt are shown in Table 1.

#### 2.1.2. Filler

The plain asphalt mastics used in this study were prepared in a 1:1 ratio of base asphalt binder and filler. The physical properties of filler are shown in Table 2.

#### 2.1.3. Fibers

**Figure 1:**
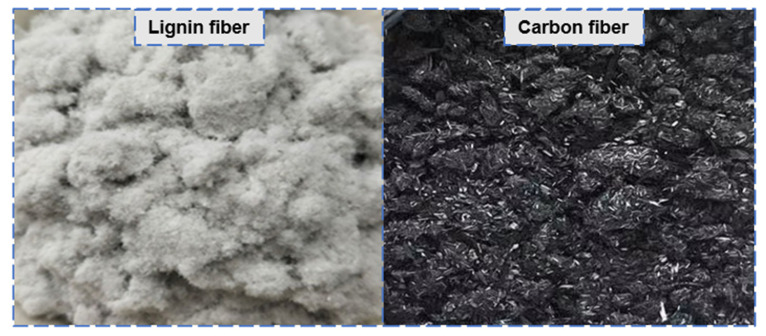
Fiber morphology.

Considering that lignin fibers tend to agglomerate in asphalt mastics with high content, the rheological performance of lignin-modified asphalt mastics at lower content was investigated. In this study, 0.5 mm carbon fiber and lignin fiber were selected as modifiers to prepare modified asphalt mastic, and the carbon fiber content was selected as 3%, 6%, and 9%, and the lignin fiber was selected as 1%, 3%, and 6% in order to adsorb enough asphalt and so that the fiber would not agglomerate. The performance of fibrous asphalt mastics at 3% and 6% content can also be compared with that of carbon fiber modified asphalt mastics and the results are equally valid. The macroscopic forms of carbon and lignin fibers are shown in Figure 1.

### 2.2. Methods

Since there is a lack of methodological specifications for assessing the performance of asphalt mastic materials, this study refers to test specifications for asphalt for testing and analysis.

#### 2.2.1. Preparation of Fiber-Asphalt Mastic

The preparation of fiber-modified asphalt binder is divided into the following four steps: firstly, fibers must be heated separately for 24 h at 105 °C to remove water from their surfaces; secondly, the stored solid asphalt (600 g) is preheated at 160 °C for 2 h to make it liquid for mixing with the filler and fiber; then, the fiber and filler are weighed in proportion and slowly added to the asphalt at 2000 r/min stirring in order to prevent the fibers from clumping; and finally, the asphalt binder is continuously stirred with the heated filler and fibers at 160 °C for about 30 min to produce a homogeneous fiber-modified asphalt mastic. There are 7 groups of research materials in this study, as shown in Table 3.

#### 2.2.2. Frequency Sweep Test

The rheological testing in this study was done based on an Anton Par MCR 102 dynamic shear rheometer (DSR) from Graz, Austria. A 25 mm parallel plate geometry with a 1 mm gap setup was used when the test temperature was above 40 °C, and a 2 mm gap geometry with an 8 mm parallel plate was used when the temperature was below 40 °C. In the present study, frequency sweep tests from 0.1 rad/s to 100 rad/s at seven temperatures of 10 °C, 20 °C, 30 °C, 40 °C, 50 °C, 60 °C, and 70 °C were selected, and the experimental results were analyzed by fitting the master curve according to the Christenson–Anderson–Marasteanu (CAM) model [26,27].

Based on the time–temperature superposition principle, the dynamic shear modulus at multiple temperatures is shifted to construct the modulus master curve, which is then matched to the Christensen–Andersen–Marastanou (CAM) model; Equation (1) illustrates the process of fitting dynamic modulus master curves:

(1) $$\left|G^{*}\right|=\frac{\left|G_{g}^{*}\right|}{{\left[1+{\left(f_{c}/f^{'}\right)}^{k}\right]}^{m/k}}$$

where |G^*^| is the dynamic shear modulus; G^*^_g_ is the glassy modulus taken as 1 GPa [28]; *f*_c_ is the crossover angular frequency; *k* and m are dimensionless shape parameters referred to as the rheological index; *f*’ is reduced angular frequency, *f*’=*φ*_T_ × *f*, where *f* is physical angular frequency; and *φ*_T_ is time–temperature shift factor fitted via a polynomial function, as shown in Equation (2):

(2) $$Log\varphi_{T}=-\frac{D_{1}\cdot \left(T-T_{0}\right)}{D_{2}+\left(T-T_{0}\right)}$$

where *T*_0_ id reference temperature, and *D*_1_ and *D*_2_ are regression coefficients.

#### 2.2.3. Linear Amplitude Sweep Test

**Figure 2:**
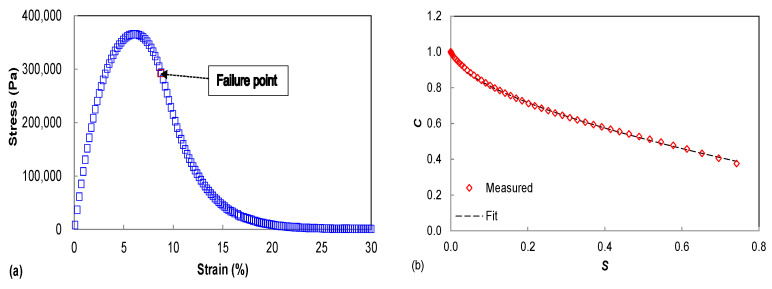
Typical fatigue model of base asphalt binder based on LAS at 20 °C (**a**) Stress–strain curve (**b**) Damage characteristic curve.

Testing was conducted using the AASHTO TP 101 linear amplitude sweep (LAS) procedure to determine the asphalt binder’s performance [29]. In this study, the theory is also applied to the fatigue performance analysis of asphalt mastic. The linear strain sweeps with amplitudes ranging from 0.1% to 30% over 5 min (hereafter referred to as LAS-5) was used in the LAS test at the desired intermediate temperature. LAS test results are shown in Figure 2. LAS test data is interpreted using the simplified-viscoelastic continuum damage (S-VECD) model developed for asphalt concrete fatigue modeling [30,31]. As the Beijing region typically experiences intermediate temperatures of around 20 °C, this study used 20 °C as the temperature for the LAS test. Taking the artificial failure definition of 35% reduction in |G^*^|·sin *δ* estimates the binder fatigue life [32].

#### 2.2.4. Multiple Sweep Recovery Test

The MSCR test based on AASHTO TO 70 [33] protocol was used to determine the asphalt binder’s capacity for permanent deformation under high-temperature circumstances. During creep-recovery loading mode, the DSR applies a lower stress level of 0.1 kPa after 90 s of recovery for ten cycles. Afterward, the stress is raised to 3.2 kPa, which is repeated continuously for another 10 cycles. Performance parameters for MSCR tests include recovery rate (*R*) and non-recoverable compliance (*J*_nr_). According to the Equations (3) and (4), the *R* and *J*_nr_ for any particular creep-recovery cycle are calculated, in which *γ*_0_ indicates the shear strain at the initiation of this cycle, *γ*_p_ represents Peak strain after creep duration of 1 s, and *γ*_n_ represents Unrecoverable strain after 9 s recovery. *τ* indicates the creep stress level in each cycle. *R* and *J*_nr_ are the average values of 10 creep-recovery modes at each strain level, respectively, thus giving four parameters *R*_0.1_, *J*_nr0.1_, *R*_3.2_ and *J*_nr3.2_. MSCR tests for all asphalt mastics were performed at 60 °C instead of the corresponding PG temperature, in order to compare the rutting resistance equally.

(3) $$R=\left(\gamma_{p}-\gamma_{nr}\right)/\left(\gamma_{p}-\gamma_{0}\right)$$

(4) $$J_{\mathrm{nr}}=\left(\gamma_{\mathrm{nr}}-\gamma_{0}\right)/\tau$$

#### 2.2.5. Bending Beam Rheometer (BBR) Test

The asphalt mastic was tested for its creep resistance at low temperatures using a bending beam rheometer. The BBR samples (125 mm × 12.5 mm × 6.25 mm) were cooled in an ethanol bath for 60 min at constant temperatures of −6 °C, −12 °C, and −18 °C, respectively. Two stainless steel supports were then used to support the beam and 100 g were loaded onto them. Stiffness was calculated as a function of time by monitoring deflection over time. An investigation was carried out at a loading time of 60 s to determine the creep stiffness (S) and creep rate (m) of the mastics. The creep stiffness (*S*) and creep rate (*m*) were used to assess the asphalt mastics’ low-temperature performance.

## 3. Results and Discussion

### 3.1. Effect of Frequencies on Modulus and Phase Angle

**Figure 3:**
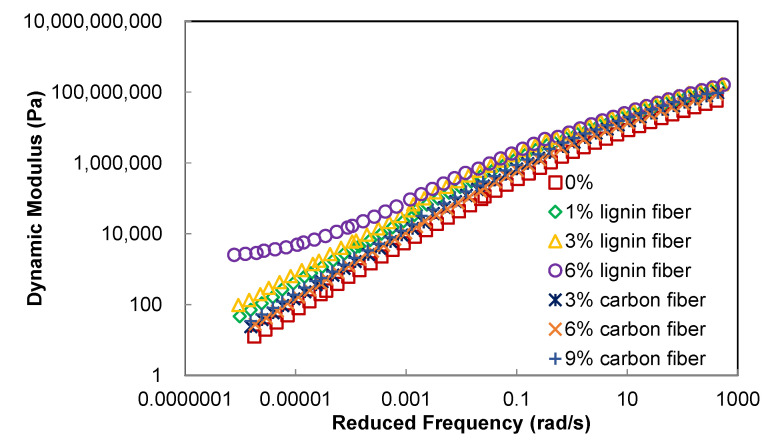
Dynamic modulus master curves.

**Figure 4:**
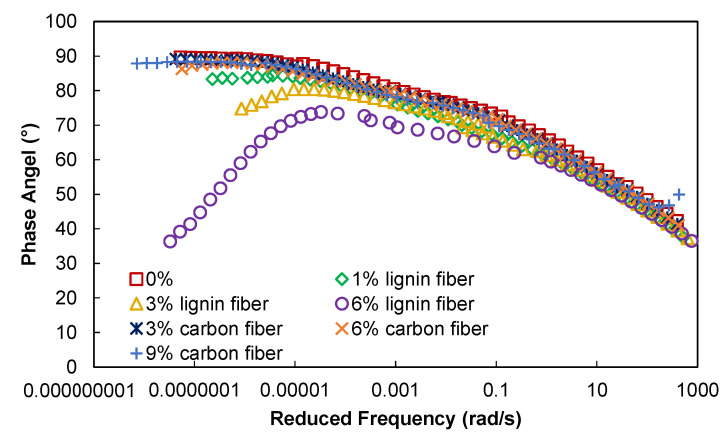
Phase angle master curves.

From Figure 3, it is clear that the dynamic shear modulus of the asphalt mastic increases with increasing loading frequency, and the logarithmic value of the modulus has a good linear relationship with the logarithmic value of the loading frequency. The reason for this relationship between modulus and loading frequency is that the greater the loading frequency, the shorter the contact time between the asphalt material and the applied loading at a single load. Furthermore, asphalt materials are viscoelastic materials whose deformation generally includes elastic deformation, recoverable viscoelastic deformation, and irrecoverable viscous deformation. The modulus of asphalt material increases with the increase of loading frequency because the higher the loading frequency, the shorter the loading action time at each cycle, and the smaller the deformation produced by asphalt material will be, resulting in the increase of the modulus. At low frequencies, the loading time becomes longer, and the deformation of the asphalt material increases, resulting in a decrease in modulus. The effect of loading frequency on the phase angle of the asphalt material has an opposite trend compared to the dynamic shear modulus, as shown in Figure 4. When the fiber content is less than 3%, the phase angle of asphalt mastic tends to decrease with increasing loading frequency, because the greater the loading frequency, the more there is an elastic deformation component in the deformation produced by each loading cycle, and thus the phase angle of asphalt material is reduced.

**Figure 5:**
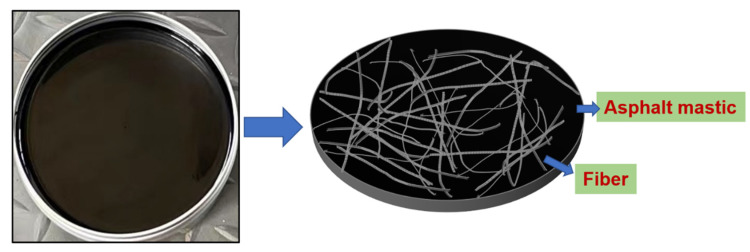
Schematic diagram of asphalt mastic sample and fiber distribution.

It can also be seen from Figure 3 that the addition of lignin fibers increases the modulus value of asphalt mastic, while the carbon fiber enhancement effect is not obvious, indicating that lignin fibers are more effective than carbon fibers in improving the stiffness and modulus of asphalt mastic. Especially at high temperatures, the stiffening effect of lignin fiber is more obvious. Compared with carbon fiber, the effect of lignin fiber on the phase angle of asphalt mastic is more apparent; whenever the fiber dose increases by 1%, it can reduce the phase angle of fiber asphalt mastic by about 5°, while when the fiber dose is higher than 3% in the high-temperature range, the phase angle of asphalt mastic shows a trend of first rising and then falling, indicating that its elastic properties first decline and then enhance. An analysis of the addition of fibers in ordinary asphalt mastic, fibers which can play the role of “macromolecular soft chain,” is shown by the schematic diagram in Figure 5. The disorderly distribution of fibers can absorb most of the concentrated stress so that the asphalt material, to withstand the load capacity while making its elastic properties, increases.

### 3.2. Effect of Temperatures on Modulus and Phase Angle

**Figure 6:**
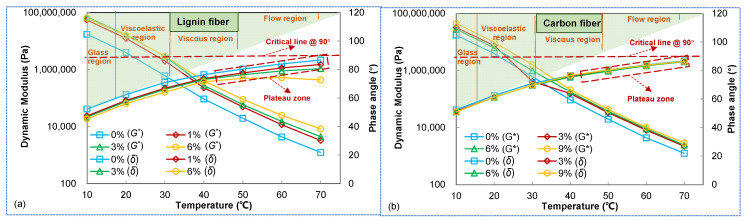
Effect of temperature on dynamic modulus and phase angle (**a**) dynamic modulus (**b**) phase angle.

The dynamic shear modulus and phase angle *δ* at each temperature at a frequency of 10 rad/s were selected for a comparative analysis in this study. Figure 6 illustrates the effects of experimental temperature on the complex shear modulus and phase angle *δ* of asphalt mastic. According to Figure 6, asphalt mastic’s dynamic shear modulus decreases exponentially as the experimental temperature increases until it finally converges when experiment temperature increases, indicating that both fiber asphalt mastic and plain asphalt mastic are sensitive to temperature; furthermore, the incorporation of fiber can substantially increase the dynamic shear modulus of asphalt mastic and make the material more resistant to stress, i.e., produce an enhancement effect. The phase angle *δ* of different types of asphalt mastic increases gradually with the increase of experimental temperature, and the phase angle of plain asphalt mastic is larger than that of fiber asphalt mastic. The larger the value of *δ*, indicating that the larger the viscous component of asphalt mastic, the more likely it is to produce high-temperature permanent deformation. Therefore, different types of asphalt mastic gradually transformed from elastic to viscous states as the experimental temperature increased, and the viscous state of plain asphalt mastic was the most obvious and most likely to produce high-temperature permanent deformation. It can be seen that the incorporation of fibers can substantially improve the high-temperature deformation resistance of asphalt mastic.

### 3.3. Initial Self-Healing Temperature

The asphalt mastics with different fiber contents were subjected to frequency sweep experiments at temperatures of 10–70 °C and frequencies in the range of 0.1–10 rad/s. The flowability of fiber asphalt mastics can be inferred from the composite viscosity and frequency, and the values of the obtained composite viscosity and frequency were fitted to a power function according to the Equation (5) to obtain the flow characteristic index *n*.

(5) $$\eta *=m{\left|w\right|}^{n-1}$$

where: *w* indicates the frequency; η^*^ indicates the composite viscosity; *m* and *n* indicate the fitting parameters. Where the fitted parameter *n* also becomes the flow characteristic index, according to which the initial self-healing temperature of the fiber asphalt mastic is analyzed and thus its self-healing ability is studied.

**Figure 7:**
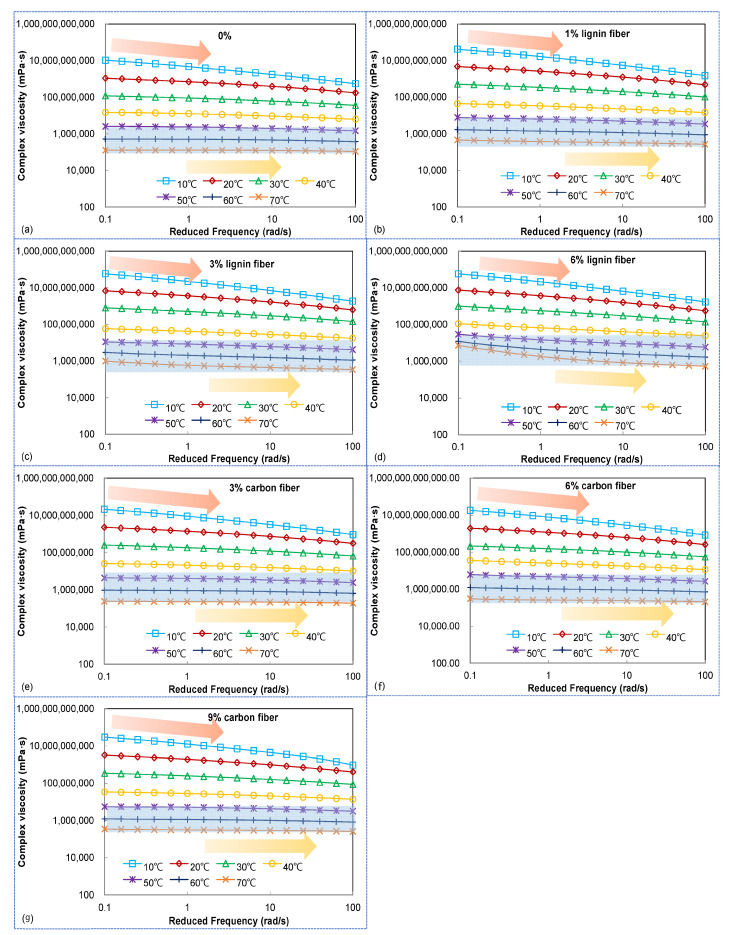
Complex viscosity of fiber modified asphalt mastics (**a**) 0% (**b**) 1% lignin fiber (**c**) 3% lignin fiber (**d**) 6% lignin fiber (**e**) 3% carbon fiber (**f**) 6% carbon fiber (**g**) 9% carbon fiber.

The test results of the composite viscosity of asphalt mastics with different fiber contents obtained from the frequency sweep test are shown in Figure 7. The composite viscosity of fiber-modified asphalt mastics will be increased more significantly with the increase of fiber contents, which is due to the formation of a space-randomly distributed network structure of fibers in the mastic impeding the flow of asphalt, which is expressed in the macroscopic increase of asphalt mastic viscosity.

Figure 7 also shows that with the increase of frequency, the composite viscosity of the fiber asphalt mastics has an obvious decreasing trend. When the temperature rises to 50 °C, the correlation between the composite viscosity and frequency becomes worse, and the viscosity curve almost becomes a horizontal straight line, indicating that at this time, the asphalt mastics compound the characteristics of the Newtonian fluid, the viscosity value is constant. After the lignin content reaches 6%, the composite viscosity at 50 °C still shows a decreasing trend with the increase of frequency, which indicates that the flow of fiber asphalt mastic is seriously hindered at higher fiber content, and it is difficult to reach the state of Newtonian fluid ultimately.

**Figure 8:**
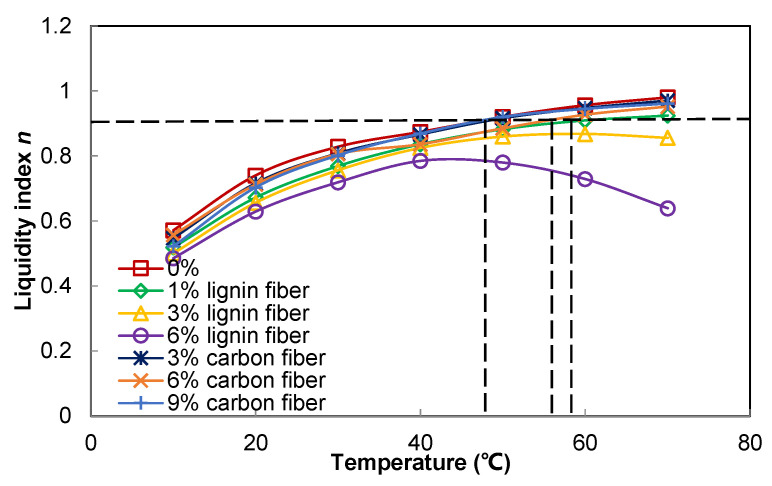
The flow characteristics index of different fiber modified asphalt mastics.

According to the results of Figure 8, the flow characteristics index of the fiber asphalt mastic was obtained from Table 4. The fitting results showed that the flow characteristics index of plain asphalt mastic and fiber-modified asphalt mastic tended to increase gradually with the increase in temperature, which indicated that the asphalt mastic gradually became a near-Newtonian fluid at higher temperatures.

The flow characteristic index of plain asphalt mastic increased from 0.571 to 0.98 in the range of 10–70 °C. The flow characteristic index of fiber asphalt mastic with lignin fibers decreased slightly, and the flow characteristic index increased from 0.519 to 0.924 and 0.5 to 0.856 and 0.485 to 0.639 in the range of 10–70 °C for 1%, 3%, and 6% of lignin fiber asphalt mastic, respectively. The carbon fiber modified asphalt mastic with 3%, 6%, and 9% of carbon fiber modified asphalt mastic increased from 0.547 to 0.97, 0.557 to 0.952, and 0.522 to 0.961 in the range of 10–70 °C, respectively, which shows that the carbon fiber modified asphalt mastic has better flow properties than the lignin modified asphalt mastic.

Figure 8 shows the tendency of the flow characteristics index with temperature for different fiber-modified asphalt mastics. As can be seen from Figure 8, fiber-modified asphalt mastic has a higher initial self-healing temperature when fibers are added, which indicates that the fiber incorporation reduces the flowability of the asphalt mastic and needs to be increased to a higher temperature to achieve the same flow state. The initial self-healing temperature of plain asphalt mastic is 46 °C at 0.9, and 57 °C at 1% lignin content. The initial self-healing temperatures for carbon fiber blending of 3%, 6%, and 9% are 47 °C, 54 °C, and 46 °C, respectively, indicating that there is a peak in the content of uniformly distributed fibers in asphalt when lower than this value. The fiber incorporation reduces the asphalt mobility and requires an increase in temperature to achieve the same flow state, and when the content is exceeded, the fibers tend to coalesce into clumps, the self-healing asphalt content increases, the asphalt mobility increases, and the temperature needed to achieve the same flow state is reduced.

As mentioned above, the initial self-healing temperature of asphalt mastic increased significantly with the increase of fiber content, indicating that the interaction between fibers affects the main factor of asphalt mastic fluidity performance when the fiber content is higher. Therefore, in the actual construction, the appropriate temperature should be set according to the different fiber types and amounts for crack self-healing, which can achieve the healing effect and reduce consumption at the same time.

### 3.4. Fatigue Performance

**Figure 9:**
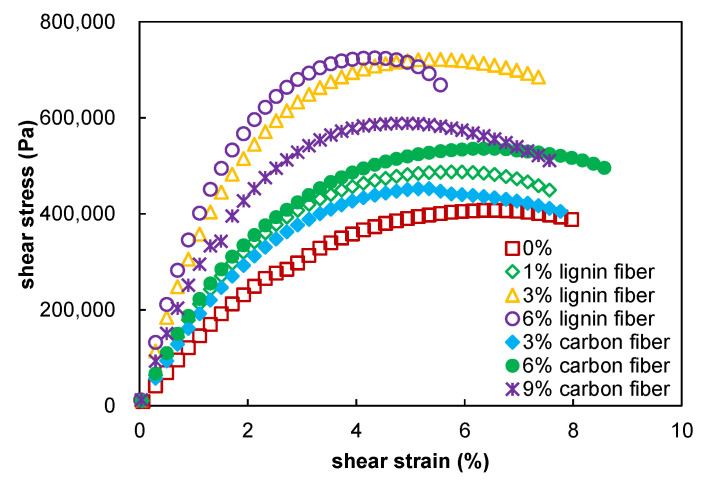
Strain–stress curves of fiber modified asphalt mastics.

This paper assumes that asphalt mastic is a homogeneous material, so the S-VECD model for asphalt is also applicable to asphalt mastic [34,35,36]. The stress–strain curves obtained based on the linear amplitude sweep test are shown in Figure 9, where the viscous damage points are identified, and the data points after the damage have been removed. The stress–strain curve of the plain asphalt mastic was lower than that of the fiber-modified asphalt mastic. The fibers increase the breaking stress while decreasing the strain, indicating that the hardener mastic consistently exhibits greater strength but has a smaller deformation limit. The highest yield strain of the asphalt material corresponds to its better elastic properties, so the increased fiber admixture adversely affects the elastic properties of the asphalt mastic. Nevertheless, the failure strain indicator only depicts the capability of the asphalt mastic under repeated loading, and it may indicate fatigue resistance in some cases. In order to obtain a more accurate fatigue evaluation, more fatigue damage and failure characteristics must be evaluated.

**Figure 10:**
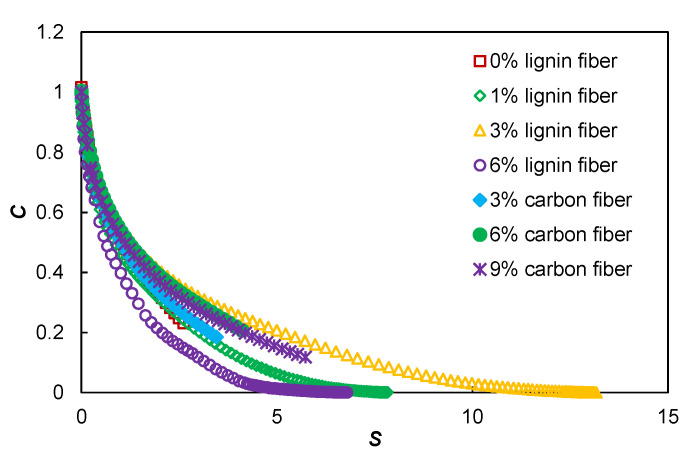
The damage characteristic curves of fiber modified asphalt mastics.

The damage characteristic curves (DCC) of plain asphalt mastic and lignin fiber-modified asphalt mastic presented in Figure 10 were calculated using the S-VECD model. The observed distinct DCCs suggest that the lignin fibers significantly affected the fatigue damage evolution of the base asphalt mastic. Using the *C* vs. *S* relationship as an input to fatigue performance prediction, the fitting results of each DCC represent the unique damage property of each asphalt mastic. The *C*(*S*) curve then gradually increases with increasing lignin content. The relevant position of the *C*(*S*) curve is mainly determined by the stiffness of the material, and considering the role of |G^*^| in the calculation of damage *S*, lower stiffness usually yields a lower curve.

**Figure 11:**
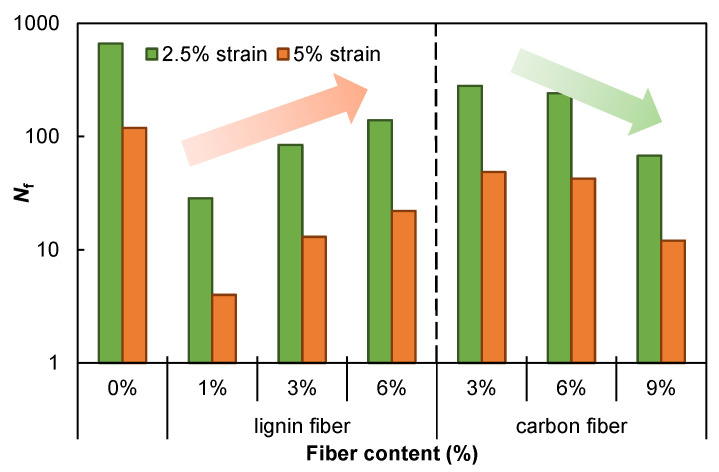
Fatigue lives of fiber modified asphalt mastics.

In this study, an artificial definition of failure, i.e., a 35% reduction in |G^*^|-sin *δ*, is adopted to estimate the fatigue life of asphalt mastic [32]. Using the calculated material properties, the fatigue life of the fiber-modified asphalt mastic was simulated and predicted under cyclic strain-controlled fatigue loading, as shown in Figure 11. Although the lignin fiber modified asphalt mastics show smaller fatigue life than plain asphalt mastic, the fiber content increase improves the fatigue lives of fiber-modified asphalt. Therefore, higher fiber content may exhibit greater fatigue life than normal asphalt mastic and is worthy of further investigation. However, carbon fiber incorporation significantly reduced the fatigue life, indicating that carbon fiber has an adverse effect on the fatigue resistance of asphalt mastic.

### 3.5. Rutting Resistance

There is a lack of tests and indicators to evaluate the high-temperature performance of asphalt mastic materials and there are limitations of using rutting factors to evaluate the high-temperature performance of asphalt mastic. Therefore, this study used the creep test to evaluate the high-temperature performance of asphalt mastic materials. The non-recoverable creep flexibility *J*_nr_ and the non-recoverable creep flexibility difference *J*_nr-diff_ were used as the evaluation indexes for the high-temperature performance of asphalt mastic.

**Figure 12:**
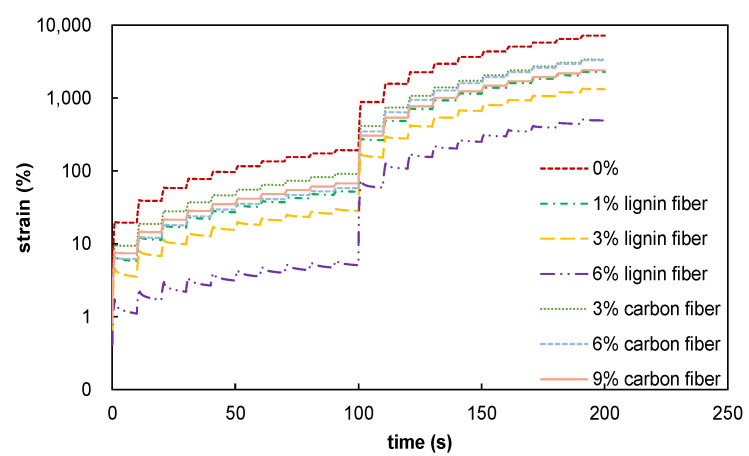
Time–strain curves of fiber-modified asphalt mastics.

**Figure 13:**
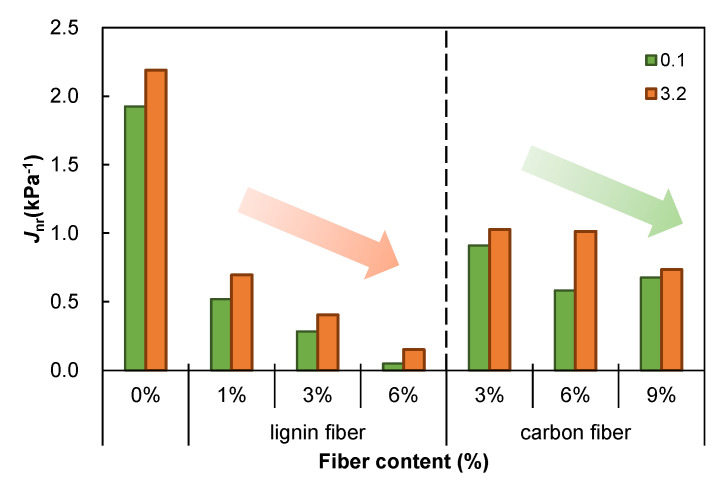
*J*_nr_ values of fiber modified asphalt mastics.

The time–strain curves measured based on the MSCR test are summarized and compared in Figure 12, which evidences that fiber-modified asphalt mastics have higher high-temperature stability than plain asphalt mastics. Increased fiber content gradually enhances fiber-modified asphalt mastic’s high-temperature stability. *J*_nr_ is used as a standard index to evaluate the irrecoverable creep flexibility of asphalt materials. Its value can more accurately reflect the high-temperature rutting resistance of asphalt materials. A lower value indicates a better high-temperature performance. As shown in Figure 13, the change pattern of *J*_nr_ is consistent with the change law of the time–strain curve, i.e., adding fiber improves its high-temperature performance. In addition, the lignin fibers have a stiffening and viscosity-enhancing effect, while the carbon fiber-modified asphalt mastic is brittle, prone to fracture, and has poor temperature resistance. Therefore, lignin fibers are chosen for the modification of asphalt mastic, which will result in a better modification effect and reduce the cost of the experiment.

**Figure 14:**
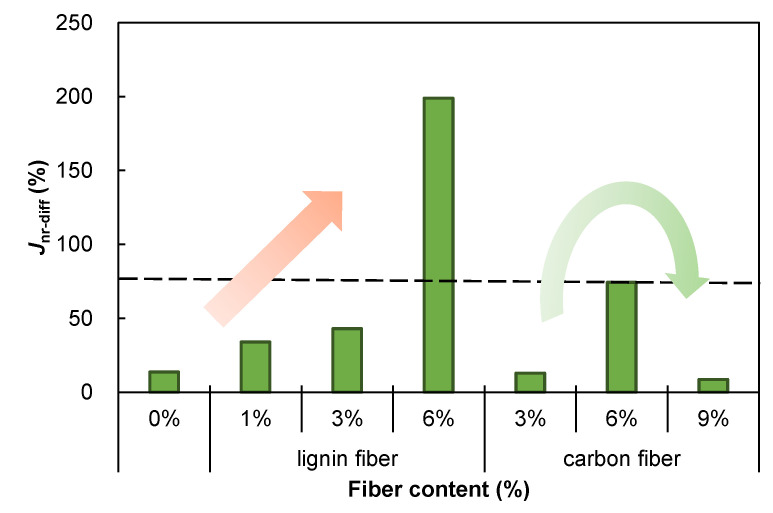
*J*_nr-diff_ of fiber modified asphalt mastics.

The results of the high-temperature creep stress sensitivity analysis of the fiber-modified asphalt mastic based on the *J*_nr-diff_ index are presented in Figure 14. As the lignin content of the modified asphalt mastic rises, the stress sensitivity of the modified asphalt mastic appears to rise sharply, especially when the lignin content reaches 6%. In asphalt mastic modified with carbon fiber, the stress sensitivity increases with fiber content and then decreases. AASHTO MP 19 [37] specifies that the limit for *J*_nr-diff_ index is 75%, which means that asphalt mastic mixed with 6% fiber has reached creep damage stage and is incompatible with the technical standards, which means that modified asphalt mastic is subject to the stress sensitivity requirements.

### 3.6. Low Temperature Cracking Resistance

**Figure 15:**
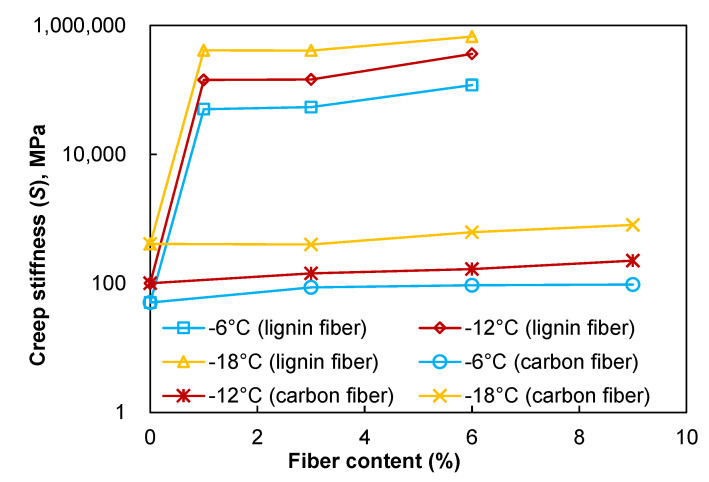
Creep stiffness of fiber modified asphalt mastics.

**Figure 16:**
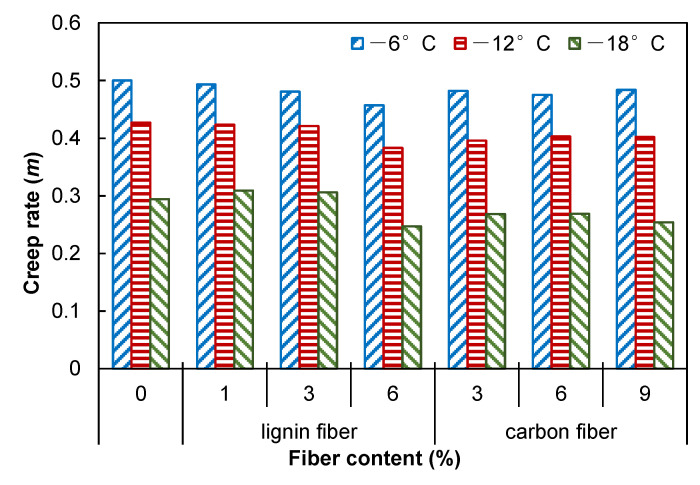
Creep rate of fiber modified asphalt mastics.

Two indicators of the BBR test: bending creep modulus of strength *S* and creep curve slope *m* (slope of the curve of strength modulus to load time) are used to evaluate the low-temperature properties of asphalt mastic. Asphalt mastic with a low *S* value is more flexible, has a greater deformation tolerance, and is more resistant to low-temperature cracking [38,39]. It can be seen from Figure 15 that the creep modulus *S* of asphalt mastic increases with the increase of fiber content. It shows that the low-temperature cracking resistance of asphalt mastic becomes worse, so the increase of fiber content is not conducive to improving the low-temperature characteristics of asphalt mastic. Meanwhile, the experimental results also reveal that the *S* value decreases rapidly with the increase in temperature, so increasing the temperature is beneficial to improving the low-temperature performance of asphalt mastic. The slope of creep curve *m* characterizes the relaxation performance of asphalt mastic. In general, increasing the value of *m* will result in faster stress release, more substantial relaxation capacity, and better crack resistance at low temperatures. Figure 16 shows the effect of fiber content on the slope of creep curve *m* of asphalt mastic. The value of the slope of creep curve *m* of asphalt mastic decreases slightly with the increase of asphalt content, but the changing trend is not apparent, which indicates that the fiber content has less effect on the stress accumulation ability of asphalt mastic, so the increase of fiber content has a negative effect on the low-temperature performance of asphalt mastic, but the effect is weak. Furthermore, asphalt mastic’s *m* value increases quickly with increasing temperature, which has a beneficial effect on crack resistance at low temperatures.

### 3.7. Four-Parameter Burgers Model Fitting Analysis

#### 3.7.1. Four-Parameter Burgers Model

**Figure 17:**
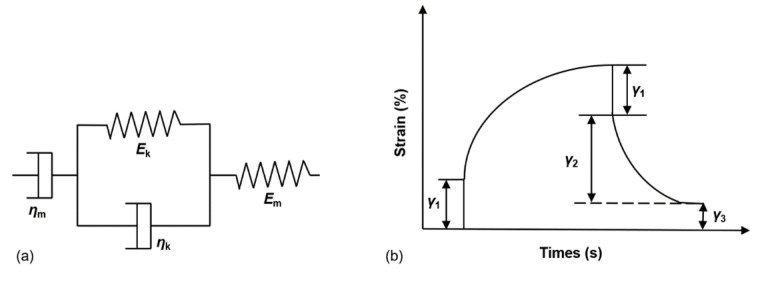
Burgers model graphs and creep curves (**a**) Burgers model (**b**) creep curves.

The four-parameter Burgers model is a widely used viscoelastic mechanics model, and it can better reflect the viscoelastic properties of asphalt-based materials. The viscoelastic model consists of a set of Maxwell models in series with a set of Kelvin models, which can respond to the instantaneous elastic strain, viscoelastic strain, and viscous strain of viscoelastic materials [40,41,42]. The Burgers model and its creep curve are shown in Figure 17. The model generally contains two equations, one is the creep loading equation with constant stress input, and the other is the stress relaxation mode equation with constant strain input, and the two equations can be obtained by Laplace transformation and inversion. The mode of creep loading with constant stress is used in this study, and its instanton equation is shown in Equation (6):

(6) $$\frac{\varepsilon (t)}{\sigma_{0}}=\frac{1}{E_{m}}+\frac{t}{\eta_{m}}+\frac{1}{E_{k}}(1-e^{-tE_{k}/\eta_{k}})$$

The creep flexibility of viscoelastic asphalt materials under creep loading *J* is generally divided into three components, as shown in Equation (7):

(7) $$J(t)=J_{e}+J_{ev}(1-e^{-t/J_{ev}})+J_{v}$$

where $J_{e}=1/E_{m}$ is the elastic flexibility; $J_{ev}=\eta_{k}/E_{k}$ is the delayed elastic flexibility or elastic flexibility; $J_{v}=t/\eta_{m}$ is the viscous flexibility.

#### 3.7.2. Viscous Part of Creep Stiffness *G*_v_

**Figure 18:**
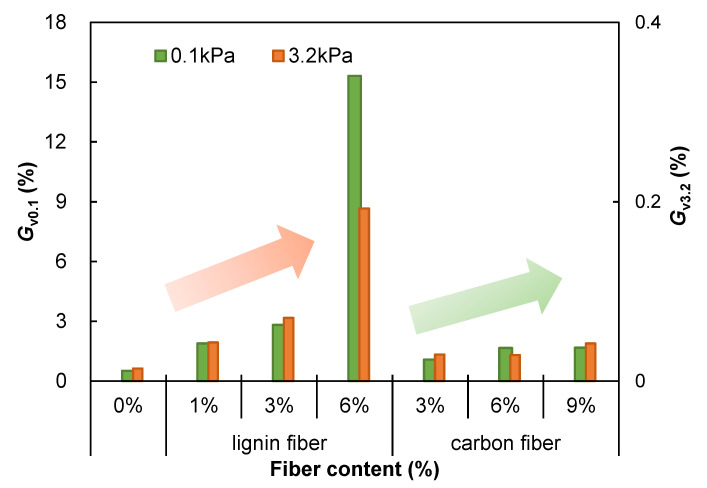
Viscous part of creep stiffness *G*_v_ of fiber modified asphalt mastics.

The NCHRP 9–10 group has proposed to evaluate the high-temperature rutting resistance of asphalt binders based on the “viscous component of creep stiffness (*G*_v_, and *G*_v_, =1/*J*_v_)”, and *G*_v_ is a parameter indicating the resistance of the asphalt binder to deformation at high temperatures [43]. The creep recovery behavior of the tested asphalt mastic samples was analyzed in this paper based on the four-parameter Burgers model, and the 10th creep-recovery cycle at two creep stress levels were selected for simulation fitting, and the *G*_v_ index of the viscous component of the creep stiffness was calculated and summarized in Figure 18. In the presence of different types of fibers, asphalt mastic’s *G*_v_ value is enhanced to some extent, giving fiber-modified asphalt mastic a significantly better rutting resistance compared to plain asphalt mastic. This is generally consistent with the *J*_nr_ index test results in Section 3.5 based on MSCR testing; therefore, the high temperature rutting resistance of the asphalt mastic evaluated by the *J*_nr_ index is consistent with the fitted analysis based on the four-parameter Burgers model.

#### 3.7.3. Model Reliability Verification

**Figure 19:**
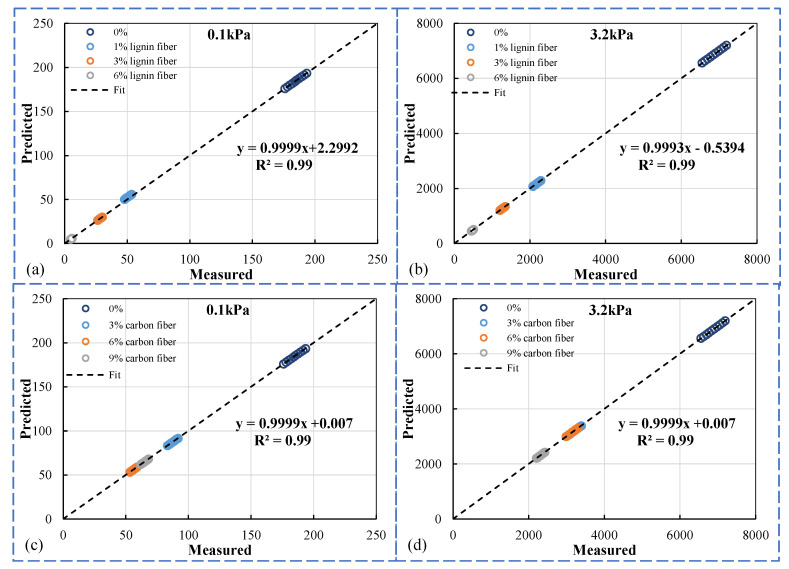
Measured values vs. Predicted values of *J*_nr_ under two stresses. (**a**) lignin fiber modified under 0.1 kPa (**b**) lignin fiber modified under 3.2 kPa (**c**) carbon fiber modified under 0.1 kPa (**d**) carbon fiber modified under 3.2 kPa.

This study was conducted by using Origin’s own formula editor to custom edit the required functions and then to fit the data, and finally the fitted parameters of the Burgers model were obtained based on the convergence of the data. Figure 19 shows the results of back-calculating the data to verify the fitness of the fitted parameters. A good correlation exists between measured and predicted values, and the correlation parameter is close to one. Therefore, it can be demonstrated that the parameters obtained from the model fitting can be used for the subsequent study of viscoelastic component analysis.

#### 3.7.4. Viscoelastic Component Comparison

**Figure 20:**
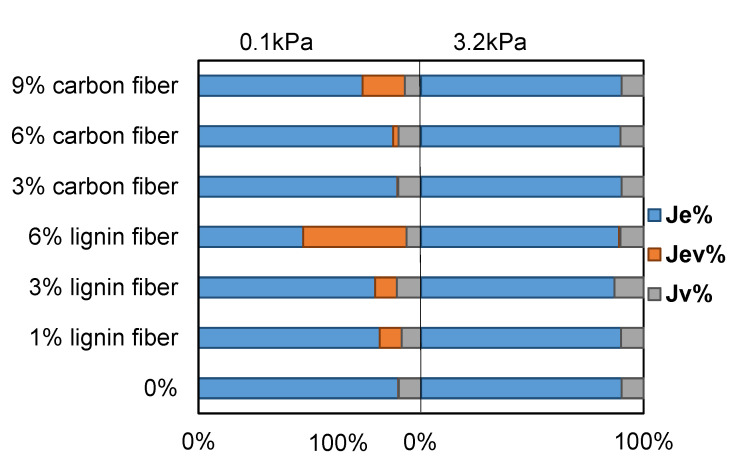
Viscoelastic component comparison of fiber modified asphalt mastics.

Based on the results of the four-parameter Burgers model fitting, a comparative analysis of the instantaneous elastic compliance, delayed elastic compliance, and viscous compliance of the asphalt during creep recovery in the MSCR test can be carried out, as shown in Figure 20. It can be seen that under the lower creep stress of 0.1 kPa, the percentage of elastic compliance and viscous compliance gradually decreases and the percentage of delayed elastic compliance gradually increases with the addition of fibers; however, under the higher creep stress of 3.2 kPa, the asphalt mastic samples basically only reflect the elastic compliance and viscous compliance, and the delayed elastic compliance generally accounts for a relatively low percentage, indicating that the creep stress level is crucial for the analysis of the viscoelastic component of the creep recovery process of fiber-modified asphalt mastic.

## 4. Conclusions

A series of studies on lignin fiber and carbon fiber-modified asphalt mastic based on DSR rheometer and BBR rheometer were conducted in this study and the following findings were obtained:

1. The addition of fibers enhances the stiffness of the asphalt, strengthens its resistance to deformation, and raises its self-healing temperature.
2. Fibers positively enhance the rutting resistance of asphalt mastics but negatively affect their fatigue resistance.
3. The low-temperature crack resistance of asphalt mastics is adversely affected by adding fibers, while the low-temperature crack resistance is positively affected by the increase in temperature.
4. Analysis of the viscoelastic component by the Burger model revealed that adding fibers increased the percentage of delayed elasticity and decreased the percentage of elastic compliance and viscous compliance of the asphalt mastic.
5. Lignin fibers are chosen for the modification of asphalt mastic, which will result in a better modification effect and reduce the cost of the experiment.
6. Natural plant fibers have a large surface area, a rough surface compared to other fibers, are highly oleophobic, and are mainly used as oil-holding, stabilizing anti-leakage, and reinforcement for asphalt mixtures. However, natural plant fibers have defects such as poor compatibility, hydrophilicity, and poor thermal stability, and the surface modification of plant fibers can be further investigated to solve these problems. Although the performance of carbon fiber is excellent, the price is too high to replace the existing engineering materials on a large scale, so how to obtain relatively low production costs has become the direction of the future development of carbon fibers. In addition, the conclusion that fiber incorporation adversely affects the low-temperature performance of asphalt binders is contrary to the fiber cracking mechanism, and perhaps fiber-aggregate interactions may change this. Therefore, it is worthwhile to follow up with more in-depth research and analyses of fiber asphalt mixtures.

## Figures and Tables

**Table 1:** Physical characteristics of base asphalt.

Properties	Testing Standards	Results
Penetration (0.1 mm) at 25 °C	JTG E20-2011/T0604	75.2
Softening point (°C)	JTG E20-2011/T0606	49.2
Ductility (cm) at 5 °C	JTG E20-2011/T0605	35.5
Viscosity (Pa·s) at 135 °C	JTG E20-2011/T0625	0.35

**Table 2:** Physical properties of filler.

Apparent Density g/cm^3^	Water Content (%)	Hydrophilic Coefficient	Appearance	Screening Test (%)		
				100	90–100	75–100
2.792	0.33	1.0	No agglomeration	100	95.8	83.1

**Table 3:** Overview of all tested asphalt mastics.

NO.	Percent Weight of Lignin Fiber (%)	Percent Weight of Carbon Fiber (%)
1	0	0
2	1	0
3	3	0
4	6	0
5	0	3
6	0	6
7	0	9

**Table 4:** Flow characteristic index of tested asphalt mastics.

T (°C)	0%	Lignin Fiber Content (%)			Carbon Fiber Content (%)		
		1%	3%	6%	3%	6%	9%
10	0.571	0.519	0.5	0.485	0.547	0.557	0.522
20	0.74	0.672	0.656	0.629	0.716	0.712	0.703
30	0.828	0.769	0.756	0.719	0.808	0.806	0.802
40	0.874	0.836	0.825	0.785	0.866	0.836	0.869
50	0.92	0.882	0.86	0.78	0.916	0.885	0.92
60	0.956	0.909	0.868	0.729	0.947	0.927	0.945
70	0.924	0.924	0.856	0.639	0.97	0.952	0.961

## Data Availability

The study did not report any data.
